# Anisotropic bias dependent transport property of defective phosphorene layer

**DOI:** 10.1038/srep12482

**Published:** 2015-07-22

**Authors:** M. Umar Farooq, Arqum Hashmi, Jisang Hong

**Affiliations:** 1 Department of Physics, Pukyong National University, Busan, Korea 608-73.

## Abstract

Phosphorene is receiving great research interests because of its peculiar physical properties. Nonetheless, no systematic studies on the transport properties modified due to defects have been performed. Here, we present the electronic band structure, defect formation energy and bias dependent transport property of various defective systems. We found that the defect formation energy is much less than that in graphene. The defect configuration strongly affects the electronic structure. The band gap vanishes in single vacancy layers, but the band gap reappears in divacancy layers. Interestingly, a single vacancy defect behaves like a p-type impurity for transport property. Unlike the common belief, we observe that the vacancy defect can contribute to greatly increasing the current. Along the zigzag direction, the current in the most stable single vacancy structure was significantly increased as compared with that found in the pristine layer. In addition, the current along the armchair direction was always greater than along the zigzag direction and we observed a strong anisotropic current ratio of armchair to zigzag direction.

Two-dimensional (2D) materials have a very large volume to surface ratio, so they display remarkable physical, chemical, mechanical and electrical properties. Owing to these properties, graphene, BN, C_4_N_3_ and MoS_2_ are receiving extensive research interest due to their unique physical properties for innovative potential device applications^1^,^2^,^3^,^4^,^5^,^6^,^7^,^8^. Very recently, a new 2D material phosphorene named after its parent black phosphorous was mechanically exfoliated by scotch tape based microcleavage method from a layered bulk black phosphorus and also a single layer phosphorene was obtained by plasma-assisted fabrication process^9^,^10^. Comparing with the graphene, the most striking difference is that the phosphorene has a direct band gap, whereas the graphene shows a zero gap at K-point^11^,^12^. Due to this semiconducting feature, the phosphorene may be superior to the graphene for device applications in many ways. In addition, few layer black phosphorene based field effect transistors have high mobility and high on/off ratio and it exhibits anisotropic optical, mechanical and electron transport properties because of its puckered geometry^9^,^13^,^14^. One can find several recent theoretical and experimental works on the black phosphorus layer^15^,^16^,^17^,^18^,^19^,^20^,^21^,^22^,^23^.

Vacancy defects in a semiconductor material can greatly change the physical properties in various ways. In most of the realistic conditions, it is hardly achievable to have a pure ideal system and the defect may naturally exist in real samples. Thus, the surface roughening and defects are major issues in the phosphorene study because it plays an important role in oxidation, which increases rapidly with time after separating a single layer from a black phosphorus^24^. The defects, besides of triggering an oxidation by itself, also play a vital role in the transport properties of any material^25^. Particularly, the influence of vacancy defects on the electrical transport property is one of the key issues in determining device performance. Usually, the vacancy defects tend to deteriorate the I–V characteristics because the vacancies behave as scattering centers for charge carriers and this results in suppression of a current. Unlike the extensive studies on the transport properties of graphene, only a few reports are available on the transport properties in phosphorene layer. Nonetheless, no systematic studies have reported how the I–V characteristics change due to defects at finite bias in phosphorene^26^. To reveal the role of defect for transport property, we will consider both pristine and the defective phosphorene layer. Moreover, the directional dependency of the I–V curve will be studied as well. If one can find a giant anisotropic behavior in the transport characteristics even in the presence of defects, this will provoke intriguing issues for device applications. Here, we will calculate I–V curve along both armchair and zigzag directions. In our work, we have investigated four types of structural defects: (a) single vacancy defects (SV) (b) divacancy defects (DVs) (c) reconstructed divacancy defects (d) Stone-Wales defects.

## Numerical Results

**Figure 1:**
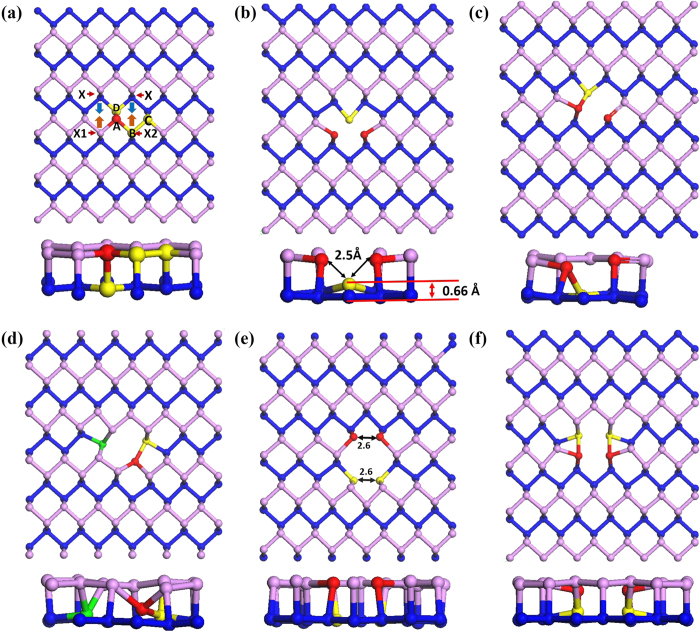
Schematic illustrations of (**a**) pristine layer with potential vacancy positions (**b**) SV1 (c) SV2 (**d**) DV1 (**e**) DV2 and (**f**) DV3.

In Fig. 1, we show pristine and five types of vacancy structures considered in our calculations. Figure 1(a) is for pristine layer and the labeling represent the potential vacant sites. We have considered two types of single vacancy (SV) structures and they are displayed in Fig. 1(b) (called SV1) and Fig. 1(c) (called SV2). For divacancy systems, we have investigated three types of configurations. Figure 1(d,e,f) represents the divacancy systems called DV1, DV2, and DV3, respectively. Since the phosphorene has puckered geometry of two layers, we indicate these two layers by pink color for upper and blue for lower layer. In SV phosphorene, we simply removed a single atom from position A and performed the structure optimization. Figure 1(b) shows the optimized structure. Due to the missing atom, a nearest lower layer atom and a pair of atoms in the upper layer moved towards the vacant position. Consequently, the atom from the lower layer moved upward about 0.66 Å and three neighboring atoms near the vacant site stretched their bond lengths about 0.1 to 0.2 Å. Their bond angles are slightly changed as well. Except for these, all other surrounding atomic positions are not noticeably changed. To discover the other possibility for a single vacancy structure, we have displaced one of the neighboring atoms towards the vacant position while the others are maintained at the original position. This breaks the even distribution of the force in the neighboring of the vacant position. Therefore, after relaxation, the displaced atom completed the third bonding with the atom from the lower layer while one phosphorous atom is left behind without making its third bonding as shown in the Fig. 1(c).

**Figure 2:**
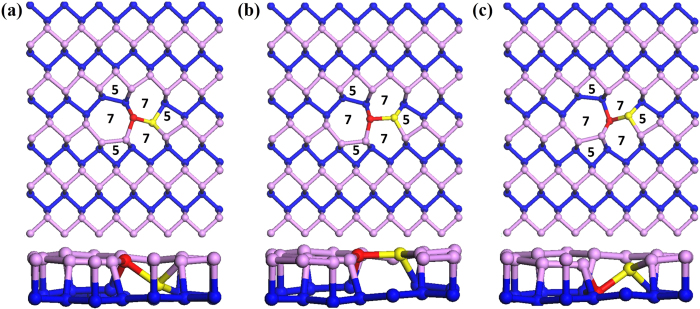
Top and side views of optimized structures for (**a**) FS1 (**b**) FS2 (**c**) FS3.

**Figure 3:**
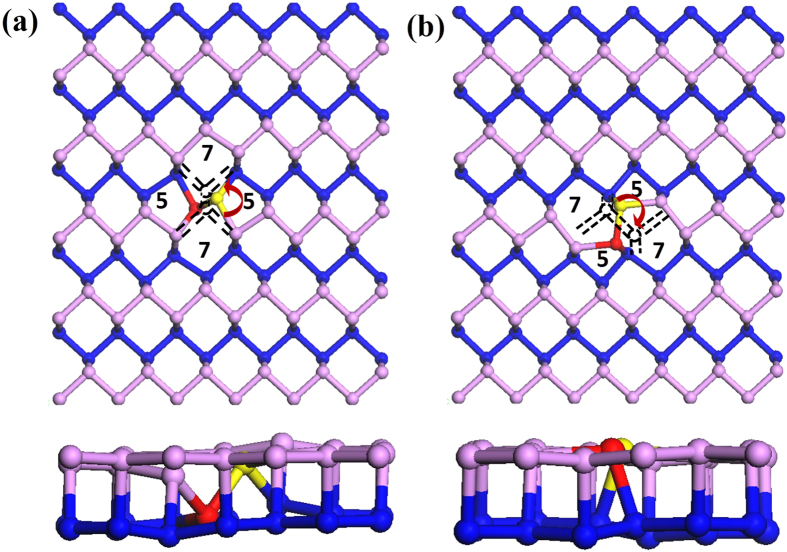
Top and side views of optimized Stone-Wales defect configurations : (**a**) SW1 (**b**) SW2. Dashed lines show the original positions of the atomic pairs.

To explore the structure of divacancy structure, we removed pairs of atoms such as AB, AC, and AD from Fig. 1(a) and displaced the surrounding atoms towards the vacant positions. When we removed the AB or AC pairs of atoms, the local geometry around the vacancy site was significantly modified after structure optimization. However, these two systems became equivalent and we presented it in Fig. 1(d). When we removed the pair AD, the atoms slightly moved to the vacant sites after performing structure optimization. Thus, the structure remained in its original shape as presented in Fig. 1(e). Another possibility was also checked. In this case, we removed the same pair AD and displaced the nearest neighbors labeled as Xs in Fig. 1(a) along the direction indicated by arrows. Then, the resultant bond lengths are not so much stretched, but the bond angles are changed as displayed in Fig. 1(f). Comprehensive studies on graphene vacancies suggested the existence of Stone-Wales defects (SWs) and transformation of simple (5–8–5) graphene divacancy in to energetically more stable 555–777 divacancy structure^27^. Thus, we have investigated 555–777 (FS) and Stone-Wales (SW) defects. Indeed, three FS vacancy defect configurations are investigated. They are presented in Fig. 2(a–c); so called FS1, FS2, and FS3. In case of FS1, the red atom is in the upper while the yellow is in the bottom layer. In FS2, both are in the same layer. In geometric point of view, the FS3 is equivalent to the FS1. Thus, we only consider FS1 and FS2 for further discussion. Stone-Wales defects do not involve any removal or addition of atoms, but they are simply created by the reconstruction of hexagonal structure into non-hexagonal rings. Four hexagons are transformed into two pentagons and two heptagons by rotation of one bonded pair of atoms. In phosphorene, two possible SW defect configurations can exist; called SW1 and SW2. The rotating pair of atoms comes from different layers in SW1 while it comes from the same layer in SW2. For the pair from the different layer, the bond rotation is adjusted at the angel of 90º as shown in Fig. 3(a). On the other hand, the bond rotation is adjusted at much shorter angel after structure relaxation in SW2 as shown in Fig. 3(b).

To find the most stable vacancy defect geometry, we have calculated the total energy difference and defect formation energies. The total energies of SV1, DV1, and SW1 structures are set to zero as a reference. Table 1 shows the calculated results. For a single vacancy structure, the SV2 geometry is more stable than the SV1 structure. The energy difference between the SV1 and the SV2 is about 300 meV/cell. For divacancy systems, the energy difference between DV1 and DV3 structures is about 120 meV/cell, and the difference between DV1 and FS1 is 151 meV/cell. Therefore, the DV1 structure becomes the most stable configuration. Nonetheless, these defect structures (DV1, DV3, and FS1) may coexist owing to various factors. However, the total energies of DV2 and FS2 are higher than 1.2 eV/cell and 0.490 eV/cell than that of DV1. Thus, the DV2 and FS2 defect geometries seem metastable states if they exist. In the case of SWs, we have observed that the SW1 is more stable than SW2. We have also calculated the vacancy defect formation energy (E_d_) defined by


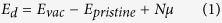


Here, 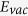 and 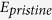 represent the total energies of the fully relaxed system with vacancy and ideal phosphorene layer, respectively. N is the number of removed atoms and μ stands for the chemical potential. We used the chemical potential of the bulk black phosphorous. Table I shows the calculated results. The formation energy of SV2 structure is smaller than SV1. The formation energies of SV1 and DV1 systems are quite close to each other. One finds the highest formation energy of 2.691 eV in the DV2 layer while the DV3 has the formation energy of 1.606 eV. As discussed earlier, both DV1 and DV3 are outcomes of fully reconstructed atomic structure in the surrounding of vacancy positions, and this feature results in lowering the total energy and formation energy. However, we have found no significant local lattice distortion in DV2 system. This caused relatively large formation energy. This reconstruction can also be seen in the FS1 and FS2 vacancy defects, but these two configurations have different formation energies. For instance, the FS1 has formation energy of 1.65 eV, and this value is very close to the DV3, whereas the FS2 has a much higher value. The defect formation energy per-atom (E_d_/N) of DV1 is 0.74 eV and that for DV3 is 0.8 eV. However, it is around 1.35 eV for DV2 and this is almost the same as that found in SV1 layer. Consequently, this implies that we can treat the DV2 as two independent SV1 type single vacancies from the nearest neighboring positions A and D in Fig. 1(a). The SWs have the lower formation energies than vacancy defect systems. In particular, the SW1 has the lowest energy. This finding suggests that the Stone-Wales type defects are more likely to exist in phosphorene. The vacancy defect formation energy in graphene was about 7.3–7.5 eV for single, from 6.4 to 8 eV for divacancies and 4.5–5.3 eV for Stone-Wales^27^. This indicates that the defect formation in the phosphorene layer will be much easier as compared with the graphene case.

**Figure 4:**
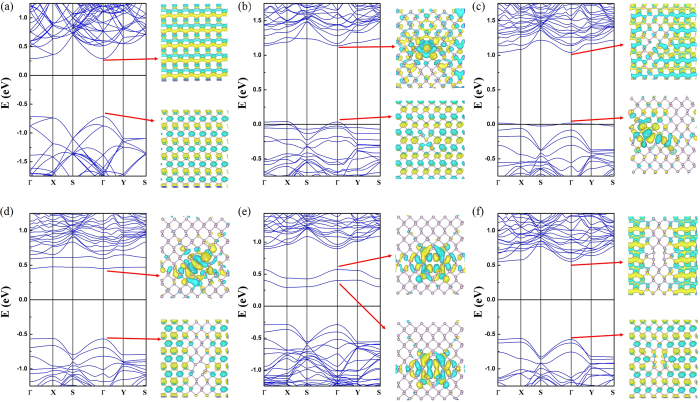
Band structures and wavefunctions at Γ point for (**a**) pristine layer (**b**) SV1 (**c**) SV2 (**d**) DV1 (**e**) DV2 and (**f**) DV3 structures.

**Figure 5:**
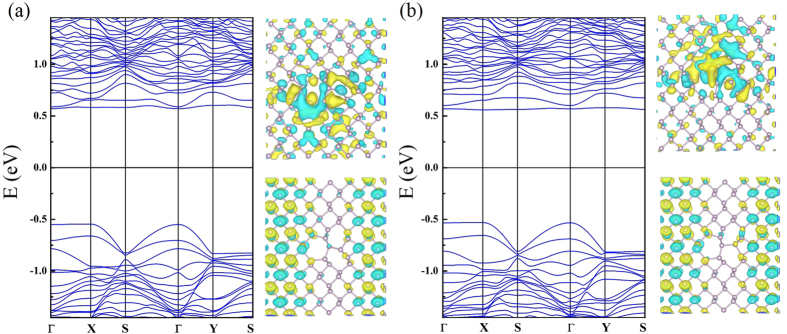
Band structures and wavefunctions of conduction band minima (up) valence band maxima (down) at Γ point for (**a**) FS1 and (**b**) FS2.

**Figure 6:**
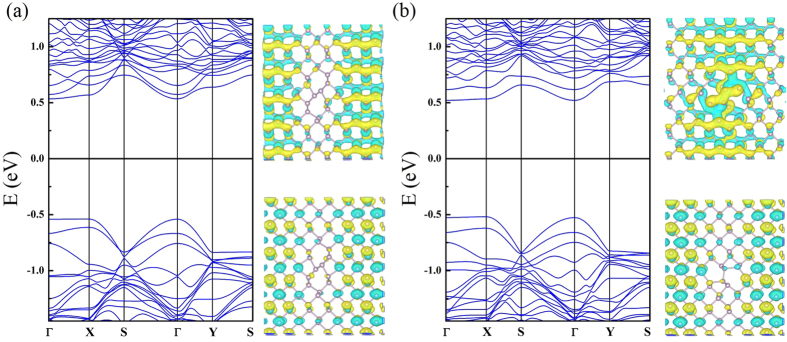
Band structures for Stone-Wales with wavefunctions of conduction band minima (up) valence band maxima (down) at Γ point for (**a**) SW1 and (**b**) SW2.

Figure 4(a) shows the band structure of the pristine phosphorene layer and we have found a semiconducting state with a direct energy gap of 1.01 eV. Figure 4 (b–c) shows the band structures of SVs. The band structure is substantially modified. First of all, the band gap disappears and the SVs layers have a metallic state because we find a defect induced states crossing the Fermi level. Furthermore, we have also observed that the wavefunction related to this state at Γ point is completely delocalized in SV1 case as shown in Fig. 4(b). In contrast, we find rather localized feature in SV2 as displayed in Fig. 4(c). Nonetheless, it still shows a delocalized character to some extent along the zigzag direction. Therefore, this band can contribute to the electrical transport. In addition, the newly found defect states in SVs are located slightly above the top of the valence band (VB). From this finding, we suggest that the SVs defects behave like a p-type impurity. In divacancy systems, the defective layer turns into a semiconductor because an energy gap reappears. Moreover, the band structures show a structural dependency of vacancy defect configuration. Figure 4(d) displays the band structure of DV1. The valence band structure does not differ from that of the pristine layer and a direct energy gap of 1.02 eV is found. This band gap is almost the same as that of the pristine layer. One can find a defect induced state. The defect state is located slightly below the bottom of the conduction band (CB). Thus, this vacancy defect may act as n-doping impurity. As one can expect from the nearly flat dispersion relation, this state is strongly localized. To confirm this, we have presented the wavefunction related to this band. The spatial localization of defect state around the vacancy is clearly observed. Therefore, this state cannot contribute to the electrical transport. Figure 4(e) shows the band structure of DV2 structure. The energy gap is suppressed to 0.58 eV and the DV2 layer has an indirect band gap. The defect states are pushed further down and these states are still localized around the vacant sites as shown by the wavefunction with the band structure in Fig. 4(d). Overall, we have found that the defect states in DV1 and DV2 cannot contribute to the electrical transport due to their localized features although they are above the Fermi level. From Fig. 2(e) for the DV3 system, we obtained a direct band gap of 1.13 eV at Γ so this vacancy increased the band gap of 0.12 eV. In addition, the calculated band structure is quite close to that of SVs, but the Fermi level is shifted and the metallic feature disappears. Figure 5(a,b) shows the band structures of FS1 and FS2. These two systems display very similar band structure with direct band gaps of 1.10 and 1.12 eV for FS1 and FS2 respectively. We have found that both systems have localized defect states at the bottom of the CB. Figure 6(a,b) shows the band structures of SW1 and SW2. The band dispersions in these two systems are very similar to each other, and the direct band gaps of 1.07 eV and 1.04 eV are observed at Γ point for SW1 and SW2, respectively.

**Figure 7:**
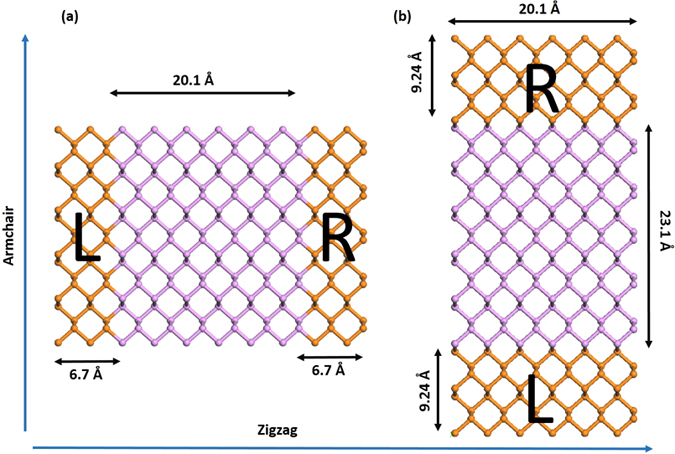
Structure diagram for transport calculations : (**a**) zigzag direction (**b**) armchair direction. Pink and orange colors represent scattering and lead part, respectively.

We now discuss the bias dependent transport properties. Figure 7 is the schematic illustration for transport calculations along both (a) zigzag and (b) armchair directions. We have calculated the transport properties using the state-of-the-art non-equilibrium Green’s function method based on DFT^28^. In the ballistic transport scheme, the current at finite bias can be obtained using the relation^29^.


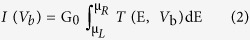


Here, the bias voltage 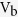 is a difference between two electrochemical potentials of left (μ_L_) and right (μ_R_) leads. T(E,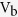) is a transmission coefficient of charge carriers transmitting through the device at a given bias voltage with the energy E and G_0_ = 2e^2^/h is a quantum conductance. Note that the chemical potential of one lead is increased by one-half of the applied bias and the other lead’s chemical potential is decreased by the same amount. Thus, the particle can contribute to the current if its energy lies between –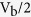 and 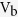/2. At zero bias, in the perfect periodic systems, both conduction and valance band regions matches perfectly. This results in perfect transmission. However, the defect breaks the periodicity and this increase the mismatch among the bands in different regions (electrodes and scattering) so that the transmission spectrum will be reduced. Higher bias also creates a mismatch between the bands, but energetic distance between the valence band of the left electrode and the CB of the right electrode decreases. As we applied the bias higher than the band gaps of the electrodes some conduction channels are created in the region around Fermi-level due to the overlapping of left electrode-valence band and right electrode-conduction band. The transmission spectrum is dependent on the relevance of the states of electrode and scattering regions and current is entirely dependent on the transmission within the integration region as in equation (2).

**Figure 8:**
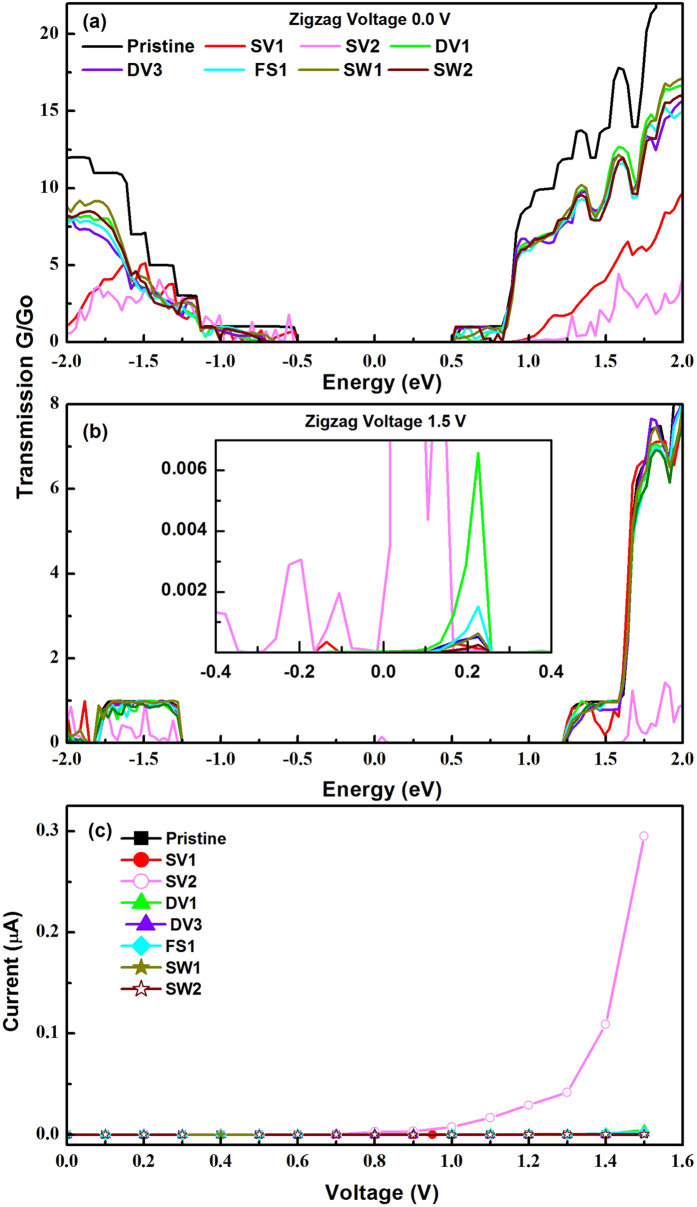
Calculated transmission coefficient along zigzag direction at (**a**) zero bias (**b**) 1.5 V (**c**) Calculated I–V curves for zigzag direction. Inset in (**b**) shows the magnified spectra. Zero is set at the center of Fermi levels of the left and right leads.

We now discuss the transport properties. In divicancy systems, we present the calculated results for DV1, DV3, and FS1 because other systems have displayed relatively large formation energies. Figure 8a,b presents the calculated transmission coefficients at zero and 1.5 volt (V) along the zigzag direction. The inset in Fig. 8(b) displays the details of the transmission coefficient within the integration region. The pristine layer had a very weak transmission coefficient and it was barely observable. The SV2 along the zigzag direction at 1.5 V has very significantly enhanced transmission coefficient implying the creation of the transmission channels along this direction. The SV1 structure shows two small peaks at approximately ±0.2 eV while all DVs have peaks only at +0.2 eV. In particular, the DV1structure had a relatively large peak. Figure 8(c) shows the calculated current with the applied bias voltage along the zigzag direction. We observed a current of 0.3 μA at 1.5 V in SV2 structure, whereas all other systems generate extremely small current because an order of nano-ampere (nA) is obtained. This feature originates from delocalized states along the zigzag direction as displayed in Fig. 4(c).

**Figure 9:**
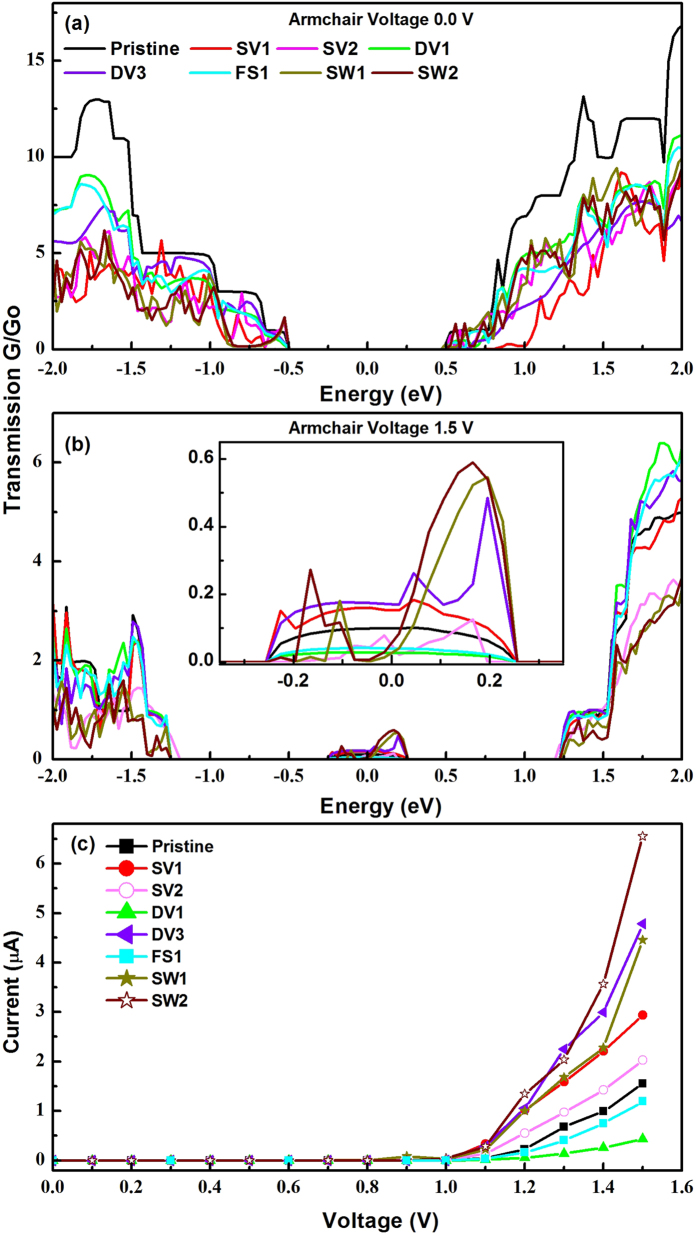
Calculated transmission coefficient along armchair direction at (**a**) zero bias (**b**) 1.5 V (**c**) Calculated I–V curves for armchair direction. Inset in (**b**) shows the magnified spectra. Zero is set at the center of Fermi levels of the left and right leads.

Figure 9(a,b) show the calculated transmission coefficients at zero and 1.5 V along the armchair. At 1.5 V one can clearly see that conducting channels are significantly increased and all systems have conducting channels within ±0.2 eV. Interestingly, we have observed that the transmission coefficient in integration region is larger in SV1 than SV2. Therefore, unlike the I–V feature along the zigzag direction, we expect that the current in SV1 structure will be larger than in SV2. Among all divacancy structures, the DV3 layer showed the largest magnitude while the transmission coefficients of DV1 and FS1 is weaker than that of the pristine layer. In Stone-Wales vacancy defects, the SW1 and SW2 have the similar transmission coefficient in integration region. We now show the I–V curve along the armchair direction in Fig. 9(c). First of all, we find a strong anisotropic behavior because the magnitude of the current is significantly enhanced along the armchair direction. In addition, the transport property shows a different tendency as the defect configuration varies. In SVs structure, the current in SV1 system is larger than found in SV2. In DV3 structure, the current is enhanced compared with that of the pristine layer, but it is suppressed in DV1 and FS1 structures. The DV1 and FS1 have localized defect states at the CB minimum. These localized states may act as scattering centers^30^. Thus, the current in DV1 and FS1 is suppressed as compared with that in the pristine layer. However, the DV3 has no such localized states near the CB minimum and VB maximum. Instead, we have found more available states due to vacancy defect and this results in enhanced current. Similarly, both SW1 and SW2 have no localized state. Consequently, the current is increased in these systems. Overall, the current along the armchair direction is dependent on the vacancy defect configuration. Indeed, in most of the conventional materials, the vacancy defects play as strong scattering centers and they tend to suppress the current. However, very interestingly the defect in the phosphorene does not show such a traditional behavior. Furthermore, the ratio of current along armchair to zigzag direction is an order of 10^3^ in pristine layer and most surprisingly this strong anisotropic feature in the presence of defects is still maintained in all system, except for SV2 system.

## Discussion

We have explored the structure, electronic band structure, and bias dependent transport properties of the defective phosphorene layer. In single vacancy structure, the SV2 structure has the lowest formation energy. In divacancy systems, the DV1 has the lowest formation energy and it was very close to that of to SV1. Comparing with the defect formation in graphene, the defect formation energy in phosphorene is five or six times smaller^18^, so the defect formation will be much easier. We have found that the electronic band structure is strongly dependent on the defect configuration. Single vacancy defect layers show metallic state and the newly found defect induced state lies slightly above the top of the VB. Therefore, we propose that the single vacancy defect behaves like a p-type impurity while the role of divacancy impurity seems to depend on their configuration. Along zigzag direction, the I–V characteristics are similar to that of the pristine layer, except for SV2. Interestingly, the current in SV2 structure was increased by approximately 10^2^ times to the current in the pristine layer. Along the armchair direction, the current in SV1, DV3 and SWs systems are significantly enhanced as compared with that of an ideal structure. However, we find rather suppressed currents in both DV1 and FS1 layers. Nonetheless, we have obtained that the current along the armchair direction is greatly larger than along the zigzag direction. In most of the materials, the vacancy defects play as scattering centers and tend to decrease the current. However, we have found an unconventional transport property because the current can be even enhanced due to vacancy defects. Furthermore, our results indicate that the anisotropic transport property is dependent on the defect configuration. The anisotropic current ratio was about 10^3^ in most of the vacancy defect systems, except for SV2 geometry. In SV2 case, the ratio was decreased to 10. Despite the suppression in anisotropic ratio, we still found large anisotropic transport property. This peculiar physical property may bring intriguing issues and will stimulate further extensive experimental studies to understand the transport properties of the defective phosphorene layer.

## Computational method

All electronic structure calculations are performed using the density functional theory (DFT) code SIESTA^31^. We have used Troullier–Martins norm-conserving pseudopotential with double-zeta basis plus polarization orbitals (DZP) for all band structure and geometry relaxation calculations^32^. Energy cutoff 300 Ry is kept throughout calculations. For electron structure calculation 9 × 7 Monkhorst–Pack k-grid scheme is used to sample the Brillion zone^33^. The generalized gradient approximation (GGA) is used by applying the Perdew−Burke−Ernzerhof (PBE) exchange-correlation functional^34^. Supercell of 6 x 5, dimensions of 20.1 Å x 23.1 Å, is used for electronic structure calculation to avoid artificial interaction from neighboring unit cell. Geometry is relaxed until the force on each atom becomes less than 0.02 eV/Å, by using the conjugate gradient method. The vacancy defect ratios are 0.8% for single and 1.67% for divacancy systems, respectively. The vacuum distance of 15 Å is applied along the perpendicular direction to the sheet surface to avoid the periodicity effect in this direction.

## Additional Information

**How to cite this article**: Umar Farooq, M. *et al.* Anisotropic bias dependent transport property of defective phosphorene layer. *Sci. Rep.* **5**, 12482; doi: 10.1038/srep12482 (2015).

## Figures and Tables

**Table 1:** Calculated Energy difference and vacancy formation energy (in eV).

Vacancy Type	Energy difference (eV)	Formation Energy (eV)
SV1	0	1.48
SV2	−0.30	1.18
DV1	0	1.49
DV2	1.20	2.69
DV3	0.12	1.61
FS-1	0.15	1.65
FS-2	0.49	1.93
SW-1	0	0.60
SW-2	0.34	0.84
